# Supplementary material for: Multi‐dataset identification of innovative feature genes and molecular mechanisms in keratoconus
Source: J Cell Mol Med. 2024 Sep 19;28(18):e70079. doi: 10.1111/jcmm.70079 (PMC11412914; doi:10.1111/jcmm.70079)
Supplement: Supplementary file 2 — Material S2. [file JCMM-28-e70079-s002.zip › 7.GSEA analysis/4.MYRF/KEGG_PENTOSE_PHOSPHATE_PATHWAY.html]

Details for gene set KEGG\_PENTOSE\_PHOSPHATE\_PATHWAY[GSEA]

|  || Dataset | MYRF.group.cls#h\_versus\_l |
| Phenotype | group.cls#h\_versus\_l |
| Upregulated in class | h |
| GeneSet | KEGG\_PENTOSE\_PHOSPHATE\_PATHWAY |
| Enrichment Score (ES) | 0.12919466 |
| Normalized Enrichment Score (NES) | 0.49699435 |
| Nominal p-value | 1.0 |
| FDR q-value | 0.9896641 |
| FWER p-Value | 1.0 |
Table: GSEA Results Summary

  

Fig 1: Enrichment plot: KEGG\_PENTOSE\_PHOSPHATE\_PATHWAY      
 Profile of the Running ES Score & Positions of GeneSet Members on the Rank Ordered List

  

| SYMBOL | TITLE | RANK IN GENE LIST | RANK METRIC SCORE | RUNNING ES | CORE ENRICHMENT || 1 | TKTL1 | na | 2822 | 0.305 | 0.0391 | Yes |
| 2 | FBP1 | na | 6755 | 0.171 | -0.0064 | Yes |
| 3 | FBP2 | na | 7413 | 0.153 | 0.0351 | Yes |
| 4 | TKTL2 | na | 8333 | 0.131 | 0.0603 | Yes |
| 5 | RPEL1 | na | 9091 | 0.114 | 0.0837 | Yes |
| 6 | G6PD | na | 9675 | 0.102 | 0.1073 | Yes |
| 7 | RBKS | na | 10850 | 0.081 | 0.1053 | Yes |
| 8 | PGLS | na | 11079 | 0.077 | 0.1292 | Yes |
| 9 | TALDO1 | na | 12681 | 0.054 | 0.1045 | No |
| 10 | DERA | na | 13617 | 0.042 | 0.0943 | No |
| 11 | PRPS1 | na | 14025 | 0.038 | 0.0975 | No |
| 12 | RPIA | na | 14576 | 0.032 | 0.0943 | No |
| 13 | PGM2 | na | 15141 | 0.026 | 0.0885 | No |
| 14 | ALDOC | na | 16531 | 0.013 | 0.0534 | No |
| 15 | H6PD | na | 16580 | 0.012 | 0.0569 | No |
| 16 | PFKL | na | 17079 | 0.008 | 0.0457 | No |
| 17 | RPE | na | 17765 | 0.002 | 0.0266 | No |
| 18 | TKT | na | 20693 | -0.030 | -0.0460 | No |
| 19 | PGD | na | 20717 | -0.030 | -0.0348 | No |
| 20 | ALDOA | na | 22404 | -0.051 | -0.0632 | No |
| 21 | PFKM | na | 23161 | -0.062 | -0.0604 | No |
| 22 | PFKP | na | 23952 | -0.075 | -0.0536 | No |
| 23 | GPI | na | 24601 | -0.086 | -0.0382 | No |
| 24 | PGM1 | na | 25162 | -0.096 | -0.0164 | No |
| 25 | PRPS2 | na | 28612 | -0.178 | -0.0453 | No |
| 26 | PRPS1L1 | na | 28842 | -0.186 | 0.0218 | No |
| 27 | ALDOB | na | 32816 | -0.374 | 0.0551 | No |
Table: GSEA details [plain text format]

  

Fig 2: KEGG\_PENTOSE\_PHOSPHATE\_PATHWAY      
 Blue-Pink O' Gram in the Space of the Analyzed GeneSet

  

Fig 3: KEGG\_PENTOSE\_PHOSPHATE\_PATHWAY: Random ES distribution      
 Gene set null distribution of ES for **KEGG\_PENTOSE\_PHOSPHATE\_PATHWAY**
